# Evidence-based design for neonatal units: a systematic review

**DOI:** 10.1186/s40748-019-0101-0

**Published:** 2019-04-30

**Authors:** N. O’Callaghan, A. Dee, R. K. Philip

**Affiliations:** 1 Mid-West Department of Public Health, HSE, Limerick, Ireland; 2 Graduate Entry Medical School (GEMS), University of Limerick, Limerick, Ireland; 3 University Maternity Hospital Limerick (UMHL), Limerick, V94 C566 Ireland

**Keywords:** Evidence-based design, Family centred care, Single family room, Open bay unit, Hospital design, Neonatal intensive care unit

## Abstract

Evidence-based design (EBD) of hospitals could significantly improve patient safety and make patient, staff and family environments healthier. This systematic review aims to determine which neonatal intensive care unit design features lead to improved neonatal, parental and staff outcomes. Medline, CINAHL, Web of Science Citation Index and Cochrane Central Register of Controlled Trials Registry, were searched in January 2017. Using combinations of the relevant key words, review was performed following the recommended guidelines for reporting systematic reviews. English language limitation was applied and term limited to 2006–2016. Included studies were assigned a grade based upon their level of evidence and critically appraised using defined tools. Data were not synthesized for meta-analysis due to nature of literature reviewed and heterogeneity. Three thousand five hundred ninety-two titles were screened with 43 full-texts assessed for eligibility. Twenty nine studies were deemed eligible for inclusion. These included 19 cohort studies, two qualitative studies, seven cross-sectional studies, and one randomised control trial. Grey literature search from guidelines, and repositories yielded an additional 10 guidelines. *‘Single family room’* (SFR) design for neonatal units is recommended. An optimally designed neonatal unit has many possible health implications, including improved breastfeeding, infection and noise control, reduced length of stay, hospitalisation rates and potentially improved neonatal morbidity and mortality. High quality, family centred care (FCC) in neonatology could be assisted through well grounded, future proofed and technology enabled design concepts that have the potential to impact upon early life development.

**Electronic supplementary material:**

The online version of this article (10.1186/s40748-019-0101-0) contains supplementary material, which is available to authorized users.

## Introduction

The last century has seen improvements in maternal and perinatal mortality with significant advances, particularly in neonatology. Although immature organ systems contribute towards morbidity, these outcomes may be compounded by unfavourable neonatal intensive care environments [1].

Recently, attention has focused upon hospital design and its effect on patient safety [2]. Similar to evidence-based medicine, evidence-based design (EBD) uses the best available information from credible research to construct patient rooms, improve lighting and air quality, reduce noise, way-finding and walking distance, promote hand-hygiene, incorporate nature and accommodate families’ needs [3]. Evidence has shown that hospital design can significantly improve patient safety [2, 4] and make patient, staff and family environments healthier [2, 5–7].

This systematic review aims to identify NICU design features which improve neonatal, parental and staff outcomes.

## Methods

This review was performed according to PRISMA guidelines for reporting on systematic reviews. Medline, CINAHL, Web of Science Citation Index and Cochrane Central Register of Controlled Trials Registry, were searched electronically in January 2017, using combinations of the relevant key words and word variants for “hospital design” and “newborn intensive care unit”. The inclusion criteria were studies written in English which evaluated NICU design features (*rather than practice*) and their impacts upon newborn infants, their families and staff, included a comparison group, and were published between January 2006 and December 2016. Grey literature was also searched, details of which are available in the addendum.

Title screening was carried out by one reviewer based on agreed, pre-piloted structured forms. Full-text articles were assessed for eligibility by two reviewers with agreement by consensus. Included studies were assigned a grade based upon their level of evidence [8] and critically appraised using a number of tools. Meta-analysis was not undertaken due to insufficient numerical data. Included studies and grey literature were divided into themes or subject areas, which are expanded upon in the results section. Further details of this and the methodology used are available in the addendum.

## Results

**Fig. 1:**
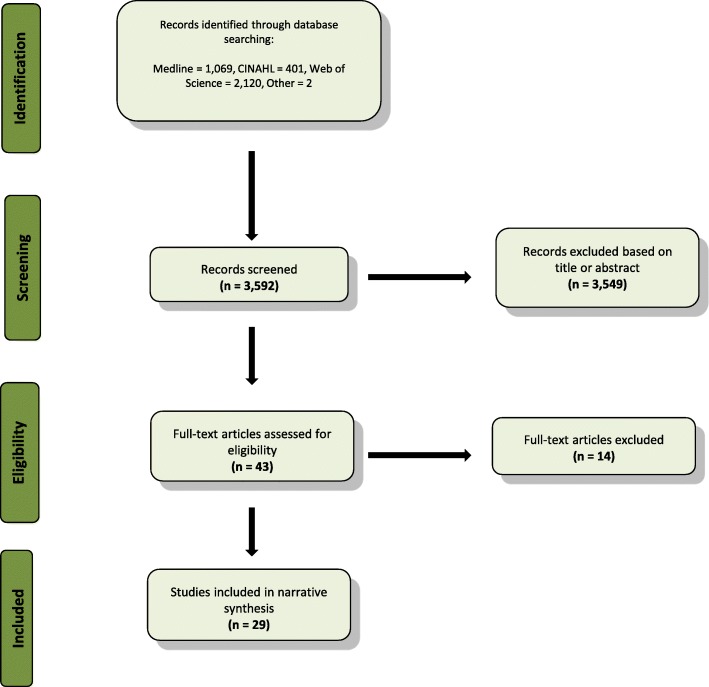
Flow diagram of results

Three thousand five hundred ninety-two titles were screened with 43 full-text articles assessed for eligibility (Fig. 1). Twenty nine articles were deemed eligible for inclusion in the review (Table 1). These included 19 cohort studies, two qualitative studies, seven cross-sectional studies, and one randomised control trial. The grey literature search resulted in the inclusion of ten guidelines (Table 2).

**Table 1:** Thematic overview of included studies by country, design and level of evidence

Study (author and date)	Country	Design	Level of Evidence
Feeding (*n* = 2)			
Steele et al. (2008) [38]	US	Cohort	IIA
Dowling et al. (2012) [40]	US	Qualitative	III
Infection (*n* = 5)			
Julian et al. (2015) [31]	US	Retrospective Cohort	IIA
Boehmer et al. (2009) [34]	US	Cohort	IIA
Von Dolinger de Brito et al. (2007) [33]	Brazil	Cohort	IIA
Domanico et al. (2011) [9]	US	Prospective Cohort	IIA
Lester et al. (2014) [11]^a^	US	Longitudinal, prospective quasi-experimental cohort	IIA
SFU versus Open-bay (*n* = 17)			
Ortenstrand et al. (2010) [13]	Sweden	Randomised Control Trial	IB
Stevens et al. (2011) [14]	US	Prospective cohort	IIA
Pineda et al. (2012) [12]	US	Quasi-experimental	IIA
Baylis et al. (2014) [17]	Sweden	Survey	III
Smith et al. (2009) [18]	US	Ergonomics/human factors evaluation	III
Bosch et al. (2012) [20]	US	Cohort	IIA
McCuskey Shepley et al. (2008) [46]	US	Survey	III
Swanson et al. (2013) [22]	US	Cohort	IIA
Allermann Beck et al. (2009) [15]	Denmark	Qualitative	III
Domanico et al. (2010) [16]	US	Prospective cohort	IIA
Stevens et al. (2010) [21]	US	Prospective cohort	IIA
Cone et al. (2010) [19]	US	Survey	III
Stevens et al. (2014) [30]	US	Sequential cohort study	IIA
Stevens et al. (2012) [10]	US	Retrospective sequential cohort	IIA
Erdeve et al. (2008) [29]	Turkey	Prospective cohort	IIA
Pineda et al. (2014) [50]	US	Prospective longitudinal cohort	IIA
Lester et al. (2014) [11]^a^	US	Longitudinal, prospective, quasi-experimental cohort	IIA
Location (*n* = 1)			
Terrin et al. (2016) [41]	Italy	Prospective Cohort	IIA
Sound (*n* = 5)			
Liu (2012) [24]	US	Survey	III
Van Enk et al. (2011) [25]	US	Survey	III
Chen et al. (2009) [26]	Taiwan	Survey	III
Stevens et al. (2007) [27]	US	Prospective cohort	IIA
Szymczak et al. (2013) [28]	US	Survey	III

^a^study repeated

**Table 2:** Included guidelines by source and country

Guideline	Source	Country
Recommended standards for newborn ICU design, 8th edition (2013)	Committee to Establish Recommended Standards for Newborn ICU Design	United States
Designing a NICU (2004)	British Association of Perinatal Medicine	United Kingdom (UK)
Infection Prevention and Control Building Guidelines for Acute Hospitals in Ireland (2008)	SARI – Health Protection Surveillance Centre (HPSC)	Ireland
Health Building Note 09–03: Neonatal Units (2013)	Department of Health	UK
National Guidance for the Control of Legionellosis in Ireland (2009)	HPSC	Ireland
Guidelines for the Prevention and Control of infection from Water Systems in Healthcare Facilities	HPSC	Ireland
Sustainable Healthcare Building Guidelines (2015)	National Health Sustainability Office	Ireland
How to specify healthy healthcare interiors (2013)	National Health Sustainability Office	Ireland
Independent Review of Incidents of *Pseudomonas aeruginosa* infection in neonatal units in Northern Ireland (2012)	The Regulation and Quality Improvement Authority	UK
Guidance for neonatal units (NNUs) (levels 1, 2 & 3), adult and paediatric intensive care units (ICUs) in Scotland to minimise the risk of Pseudomonas aeruginosa infection from water	Health Protection Scotland	UK

### Quality of included evidence

Studies tended to be observational and carried out in a single facility and consequently sample sizes were small. When outcomes such as mortality were assessed, the numbers were further reduced. Mortality was not often a primary outcome of these studies. Very few experimental trials were found. Efficacy in enhancing patient care is multivariate and it was difficult to establish causal relationships with any certainty. However, it must be accepted that experimental study designs may not be appropriate in this context and the evidence presented is the best possibly available for this research question.

Limited information on methodology was provided in the guidelines which were included, which hindered their critical appraisal. However, particularly in the case of US guidelines, these have been adapted internationally and adopted by many groups. Also, expert guidance based upon recommendations of those who work in this field is important, however not taken into account in a systematic review.

### Single family rooms versus open-bay units

#### Infant outcomes

Open-bay NICUs have the advantage of developing communication and interaction with medical staff and nurses and the ability to monitor multiple infants simultaneously. Single family rooms (SFR) were noted to improve sleep, increase privacy and parental involvement [9] and assist with infection control and noise limitation [9]. Infants were found to have fewer apnoeic events, reduced nosocomial sepsis and mortality as well as improved neonatal nutritional outcomes [10] and earlier transition to enteral feeding [9]. They have not been associated with an increased risk to patients [10].

Very low birth weight (VLBW) infants (< 1500 g) cared for in the SFR NICU weighed more on discharge, had greater rate of weight gain, needed less medical procedures, had a lower gestational age at full enteral feeding and less sepsis [11]. They showed better attention, had less hypertonicity, lethargy, pain and physiologic stress [11].

In contrast to the above studies Pineda et al expressed concerns that environmental sound and language exposure in single rooms may be reduced to levels that are detrimental to child development, with diminution of normal hemispheric asymmetry, lower language scores and a trend towards lower motor scores by two years [12]. Relatively low rates of parental visitation and holding with skin-to-skin interaction may have affected the generalizability of findings in this study.

#### Length of stay

SFRs have been noted to reduce length of hospital stay and rehospitalisation [9]. Providing “family centred care” (where parents stay overnight in the hospital) has significantly reduced length of stay (LOS) from a mean of 32.8 days in standard care (with limited opportunities for parental stay overnight) to 27.4 days in family centred care (*p* = 0.05) [13]. The authors postulated this reduction in LOS occurred as parents who spend most of their time with their newborn may have a greater opportunity to interpret and act on signs of distress and other needs of the infant compared to NICU staff who may have more than one infant under their care. In FCC units parents quickly became primary care givers and the greater continuity of care could possibly have contributed to more individualised care.

#### Parental satisfaction

When SFR and open-bay NICUs were compared for parental experiences, the SFR design resulted in greater parental satisfaction with care received [10], particularly with the environment, which was more conducive to family-centred care [14]. Premature infants cared for in single rooms experienced significantly more hours of visitation in the first two weeks of life and in weeks three and four. However, more stress has been reported by mothers in single rooms [12]. Smaller rooms where the number of infants were limited to one or two, provided space for parents to come to terms with their situation and to start the bonding process [15]. In one instance open-bay units were felt to be more conducive to social interaction with other parents [16]. However, when LOS increased parents were more appreciative of the comfort, privacy and environmental control aspects of SFRs. Those familiar with both showed a strong preference for SFRs which were felt to be preferable regarding issues of environmental control, privacy for bonding with the infant and breastfeeding [16].

The design and practices of the NICU has been found to dictate when parents first interact with their infants [17]. In general, parents who were facilitated to stay 24/7 in a unit experienced many “first moments” earlier [17].

#### Staff perceptions

Higher staff satisfaction scores for quality of physical [18, 19] or work environment [20, 21], patient care, job quality in the NICU [18, 21], health and safety [20–22], security [21], interaction with technology [18, 21] and overall satisfaction were noted for the SFR [10, 21]. Following the transition to an SFR model staff reported improved satisfaction [20, 23] and communication [20] as well as a reduction in isolation [22]. SFR design was felt to be better for patient therapy [19, 20] and recovery as well as their overall development [20, 22], including brain development [20]. The new unit (SFR) was also perceived as quieter and with lower perceptions of fatigue [20] and stress [19, 20, 23].

In contrast, Domanico et al raised concerns regarding SFR design. Early detection of medical crises (reflecting staff interaction) and adequate patient care was felt to be compromised in the SFR. However, the reduced mortality and length of stay in the SFR in this particular study did not support this perception [16]. Quality of team interaction was also noted to be initially poor [22] or show significantly decline [18]. This finding was not sustained in all instances [22]. Appropriate use of virtual audio-visual technology was suggested to improve staff visibility of others in the NICU [18]. A greater personnel need was also felt to exist with SFR use [10].

#### Sound, light, temperature and humidity

The degree of environmental control of sound and light was enhanced in SFR NICUs [10]. Median sound levels were significantly lower in the single-room or enclosed space NICU design compared to the open- bay models in four studies [24–27]. Although Liu et al. did note that when high frequency oscillatory ventilation (HFOV) was used similar measures were observed between the two units [24].

In contrast, Szymczak et al. found no statistically significant difference in sound level variance, nor percent time with peak sound variance in single-room and open-ward designs [28]. However, single-room design may offer significantly more time at lower noise levels as time below 0.05 standard deviations was higher in the single-room NICU [28].

Contrasting results were found for light level measurements. One study found that mean light levels were higher in the single (private) room design, due of the increased number of windows [25] and another recorded lower median levels of minimum and maximum illumination in the SFR NICU [27]. Low level of illumination favoured by nurses in the SFR has also been highlighted [10].

Temperature and humidity were assessed in only one study which found the single (private) room environment was cooler (two degrees), with greater temperature stability [25]. Mean humidity readings in the two environments were the same, but again humidity levels in SFR were more stable [25].

Specific acoustic and illumination guidance can be found in Additional file 1: Tables S1 and S2 in the addendum.

#### Cost

Providing family-centred care in SFR in the NICU has been found to result in fewer acute care visits, phone consultations and rehospitalizations when compared to those cared for in traditional open plan units [29]. When compared to open-bay units, care was provided in single-room NICUs at no additional cost [30] or lower costs [10].

#### Infection prevention and control

##### Single family rooms versus open-bay units

Studies examining infection control in SFR and open NICUs have shown mixed results. Incidence of nosocomial sepsis in SFRs has been shown by Domanico et al. to reduce to almost half that seen in an open unit [9]. Whereas, Julian et al., comparing MRSA colonisation, found that colonisation was impacted by hand-hygiene compliance regardless of room configuration [31]. It is also recommended that newly built acute hospital inpatient accommodation should be comprised of 100% single rooms [32].

##### Airborne infection

Regardless of overall NICU room configuration, an expert group in the US recommend that a negative pressure airborne infection isolation room, with a clear floor space of 14 m^2^, containing hand-washing facilities, space for storage, means of emergency communication and self-closing doors should be provided (41).

##### Hand-washing

Two studies demonstrated significantly increased rates of nosocomial infection when infants were moved to less spacious, temporary NICUs and subsequently decreased when infants were moved to a newly constructed facility with improved sink-to-bed ratios [11, 33]. In one further cohort study conducted as part of a *Salmonella* outbreak in a Tennessee NICU, a high number of inpatients were believed to have resulted in reduced attention to infection control procedures [34]. The inaccessibility of hand sinks was also felt to impede adequate hand-washing [34]. Several sink design specifications are available to view in Additional file 1: Table S3 in the addendum.

##### Water safety

Prevention and control of *Pseudomonas aeruginosa* and *Legionnella* in NICUs is important. Those designing or renovating NICUs should carefully consider water safety in healthcare buildings, water safety plans as well as the materials, fixtures and fittings which will be used [35–37]. Specific water safety recommendations which could be incorporated into a new building can be viewed in Additional file 1: Table S4 in the addendum.

#### Feeding facilities

Infant formula, when prepared at the bedside, was shown by Steele et al. to be 24 times more likely to be contaminated than those prepared in a centralised feeding preparation room [38]. Space for preparation and storage of formula distant from the bedside is recommended [39].

SFR design has resulted in more mothers sustaining lactation and more infants discharged with successful breastfeeding [9]. In contrast SFR design has also not been shown to increase breastfeeding duration by mothers of hospitalised preterm infants [40]. This study was underpowered, which perhaps contributed to the non-significance of findings. Participating mothers did express preference for pumping in their own homes due to enhanced privacy and environmental control [40].

#### NICU location in relation to other departments

Co-location of delivery rooms and the NICU has resulted in the reduction of moderate hypothermia and morbidity [41]. It is recommended that the NICU should be a distinct and controlled area immediately adjacent to the labour suite and rooms specified for operative deliveries [42–44].

#### Support areas

Several support areas are recommended. These include: clinical support areas, located as close as possible to clinical care areas [44]; a clerical area, located near the entrance to the NICU; one or more staff work areas each serving 8 to 16 beds [39]; staff support space, which may account for at least one-third of the floor space of the entire unit [39]; and family and infant room(s) should be provided for transitional care within or immediately adjacent to the NICU to allow those families who wish to stay with their infants the opportunity to do so [39, 42].

Further detailed specifications for these areas can be viewed in Additional file 1: Table S5 in the addendum. Also included in the addendum are design specifications for space requirements, enhancing unit security, finishes and measures to improve the NICU sustainability (Additional file 1: Tables S6 to S9).

## Discussion

This systematic review was set out to determine what NICU design elements lead to better neonatal, staff or parental outcomes.

Evidence suggests that SFR’s have improved privacy and sleep [45] infection control [9, 45], noise control [14, 45], wider environmental control [14], parental involvement and satisfaction [12, 45], reduced length of stay [9, 45], reduction in hospitalisation [45], fewer apnoeic events [9], improved mortality [9] and increased breastfeeding [9]. Staff preferences appear to tend toward SFR with some studies showing reduced stress in these settings [19, 46] although this was not replicated in all studies [12]. Concerns have been voiced over increased personnel need [45] compromised early detection of crises [16] and reduced staff interaction with the SFR design [18]. However, other studies have shown reductions in staff stress and fatigue and refutations to claims of staff isolation [20]. Although, in general evidence supports the use of SFR’s, one aspect of their use which showed mixed results was the impact such designs had upon neurodevelopmental outcomes. Research into this area is at an early stage and further studies are required.

Infection prevention and control is especially important in NICU settings where critically ill babies are at increased risk of hospital-acquired infection due to their immunological immaturity and the increased number of invasive procedures [33]. Most evidence for infection control focuses on creating an atmosphere which promotes hand-hygiene, with every infant bed, within six metres of a hands-free hand washing station [39]. Indeed two studies highlighted an increase in infection rates in settings where there was a lower sink-to-bed ratio and a third linked the inaccessibility of hand sinks to a *Salmonella* outbreak. The single room NICU is touted as a strategy which addresses environmental concerns and reduces iatrogenic effects by reducing the risk of infection and stress on preterm infants [11]. This hypothesis is supported by one study which noted a halving of the incidence of nosocomial infection when a SFR setting was compared to an open bay unit. *Pseudomonas* infection also poses a risk in NICUs. This may be offset by the detailed water safety advice mentioned previously.

Hospitals play an important role in health promotion and an environment supportive of breastfeeding is highly desirable. This is especially the case in the NICU setting where breastfeeding is of such importance to preterm population in reducing necrotising enterocolitis and sepsis. Limited evidence suggests environmental control and privacy is desirable. Given the premature population and requirement for expressed breast milk, if single patient rooms were unavailable privacy and maternal comfort could aid pumping and sustainability of breastfeeding.

Even though none of the eligible studies included in our systematic review addressed the concept of ‘blended design’ neonatal units; this practical approach perhaps optimise the available footprint and merges an open-bay (often pre-existing) design with designated SFR areas. Often such an innovative approach enhances clinical effectiveness at a reduced initial capital cost or renovation cost and ‘adapts’ a traditional open-bay unit to offer FCC. In response to a new transformational design of NICU, healthcare practitioners could develop new practices and this could also influence outcomes [47].

### Limitations

This systematic review was carried out with some shortcomings. Included studies had certain inherent limitations, as detailed previously. English language restrictions were applied, meaning some studies may have been omitted from the review. We have restricted the review period commencement from 2006, thus not including literature prior to that. Bias inherent to the individual studies would be reflected in our analysis. We did not progress with a meta-analysis considering the wide heterogeneity and variability of the studies, wide variations in the primary aims of the studies included, inclusion of both quantitative and qualitative studies as well as our inclusion of grey literature and guidelines in the analysis. We could not register our systematic review with PROSPERO as it was conducted as ‘part of the best evidence gathering process’ to design and construct a New Maternity Hospital with Neonatal Unit attached to University Hospital Limerick and the timelines preceded our study registration.

## Conclusions and recommendations

An optimally designed NICU has many possible health implications, including improved breastfeeding rates, infection and noise control, reduced length of stay and hospitalisations and potentially improved neonatal morbidity and mortality. The impact of early life development on later child health and development is well recognised [48]. NICU is the first extra-uterine setting for an increasing number of premature babies [1]. Preliminary evidence suggests that the NICU design may influence environmental exposures during a crucial period of brain development which can lead to long-term health implications. A well designed NICU has the potential to improve developmental outcomes and reduce chronic illness [49].

‘Single family room’ design for neonatal units is recommended. Careful consideration should also be given to infection prevention and control, including sink frequency and positioning, water safety features and airborne isolation facilities. Finishes used should have acoustic and illuminative suitability, as well as allowing for infection prevention and where possible, be environmentally sustainable. Support areas for families, staff and clinical activity are also important, as is the need to support mothers in breastfeeding.

Nature of the topic poses inherent limitations for conduct of randomized trials; however observational studies using standardised methodologies could add further evidence. Health service planners and design teams should be equipped with the evidence-base for positive design features that would impact the care of newborn infants, support to the caring families and wellbeing of the staff. High quality, family centred neonatal care could be supported through a well grounded, technology enabled and future proofed design concepts.

Further detailed recommendations are available in the addendum.

## Additional file

Additional file 1: **Table S1.** Design specifications for an optimum acoustic environment. **Table S2**. Design specifications for optimum lighting. **Table S3.** Recommended sink design specifications. **Table S4.** Design features to enhance water safety. **Table S5.** Design specifications for clinical, staff and family support areas. **Table S6.** Recommended space requirements for the NICU. **Table S7.** Design specifications to ensure NICU security. **Table S8.** Design specifications for NICU finishes. **Table S9.** Design specifications to improve building sustainability. (DOCX 72 kb)

## Abbreviations

CINHL: Cumulative index of nursing and allied health literature
GEMS: Graduate entry medical school
HFOV: High frequency oscillatory ventilation
HPSC: Health protection surveillance centre
HSE: Health service executive
ICU: Intensive care unit
LOS: Length of stay
MRSA: Methicillin resistant *Staphylococcus aureus*
NICU: Neonatal intensive care unit
PRISMA: Preferred reporting items for systematic reviews and meta-analyses
PROSPERO: International prospective register for systematic reviews
SARI: Strategy for control of antimicrobial resistance in Ireland
SFR: Single family room
UMHL: University Maternity Hospital Limerick
VLBW: Very low birth weight
