# Supplementary material for: Evidence-based design for neonatal units: a systematic review
Source: Matern Health Neonatol Perinatol. 2019 Apr 30;5:6. doi: 10.1186/s40748-019-0101-0 (PMC6492317; doi:10.1186/s40748-019-0101-0)
Supplement: Supplementary file 1 — Table S1. Design specifications for an optimum acoustic environment. Table S2. Design specifications for optimum lighting. Table S3. Recommended sink design specifications. Table S4. Design features to enhance water safety. Table S5. Design specifications for clinical, staff and family support areas. Table S6. Recommended space requirements for the NICU. Table S7. Design specifications to ensure NICU security. Table S8. Design specifications for NICU finishes. Table S9. Design specifications to improve building sustainability. (DOCX 72 kb) [file 40748_2019_101_MOESM1_ESM.docx]

# Addendum

# Methodology

The following research question was developed:

Are there any design aspects (as opposed to staff practices) in the neonatal intensive care unit (NICU) that relate to improved outcomes?

Search terms were identified from relevant papers. Embase was used to search for MeSH and synonyms. Boolean search modes were used. Three online databases (Medline, CINAHL and Web of Science) were searched using Boolean search modes with the terms shown in Table 1. The Cochrane Central Register of Controlled Trials was also searched.

Table 1 Search terms

| “facility design” OR “health care facility design” OR “health facility design” OR “hospital architecture” OR “architecture” OR “hospital design and construction” OR “hospital design” OR “design*” OR “construction*” OR “layout” OR “planning” |
| --- |
| AND |
| “intensive care unit, newborn” OR “intensive care units, newborn” OR “Intensive care units, neonatal” OR “intensive care unit, neonatal” OR “intensive care, neonatal” OR “neonatal intensive care” OR “newborn intensive care unit” OR “newborn intensive care units” OR “newborn intensive care units” |

Ten-year publication limits (2006 and 2016) were applied, as well as English language limits and restrictions to human research. To reduce duplication Medline articles were excluded from the CINAHL search as this database was searched separately.

Inclusion criteria were all studies written in English which evaluated NICU design features (rather than practice) and their impacts upon neonates, their families or staff, included a comparison group and were published between January 2006 and December 2016.

## Grey literature search

Six international databases of national guidelines from English-speaking developed regions were searched (Table 2). International paediatric and obstetric and gynaecological training bodies and colleges’ websites were also searched for any pertinent guidance.

Table 2 International guideline databases and societies and training bodies’ webpages searched

| International Guideline Databases | Societies and Training Bodies |
| --- | --- |
| United States National Guideline Clearing House | **Royal College of Obstetricians and Gynaecologists** |
| National Institute for Health and Care Excellence | Royal College of Paediatric and Child Health |
| Scottish Intercollegiate Guideline Network (SIGN) | **Institute of Obstetricians and Gynaecologists** |
| Canadian Medical Association’s Infobase | Royal Australia and New Zealand College of Obstetricians and Gynaecologists |
| New Zealand Guideline Group | **Australian Paediatric Society** |
| Australian National Health and Research Council | Royal Australasian College of Physicians |
|  | **American Congress of Obstetricians and Gynaecologists** |
|  | Society of Obstetricians and Gynaecologists of Canada |
|  | **Canadian Paediatric Society** |
|  | International Federation of Gynecology and Obstetrics |

Any pertinent Department of Health guidance in Ireland or the United Kingdom was sought. The Health Protection Surveillance Centre website was searched for any relevant guidance on building design and infection control.

A visit to a NICU was undertaken. Medical and nursing staff were consulted about NICU design and what they would wish to see in a new unit.

## Conduct of the review

The screening of titles was carried out by the first author. Full-text articles were assessed for eligibility by two reviewers with agreement by consensus. Included studies were assigned a grade based upon their level of evidence.^(1)^

Guidelines were critically appraised using Appraisal of Guidelines for Research and Evaluation (AGREE II) criteria for appraisal of guidelines. Studies were critically appraised using several tools (Table 3).

Table 3 Critical appraisal tools by study design

| Study Design | Critical Appraisal Tool |
| --- | --- |
| Systematic Review | Critical Appraisal Skills Programme (CASP) Systematic Review Checklist |
| Randomised Control Trial | Critical Appraisal Skills Programme (CASP) Randomised Control Trial Checklist |
| Cohort | Newcastle-Ottawa Scale for Assessing the Quality of Nonrandomised Studies in Meta-analyses |
| Cross-sectional | Joanna Briggs Institute Checklist for Analytical Cross-Sectional Studies |
| Qualitative | Critical Appraisal Skills Programme (CASP) Qualitative Checklist |

## Data extraction

After the search, studies which reported on the impact of physical design features were grouped into nine themes to determine the advantages and drawbacks in terms of outcomes for infants, parents and staff.

# Findings

Table S1 Design specifications for an optimum acoustic environment

| 1. Recommended acoustic parameters:   Infant rooms: Continuous background and operation sounds not exceeding an hourly L_eq_ of 45dB (40 dB in UK^(2)^) and an hourly L_10_^[[1]](#footnote-1)^ of 50dB (both A weighted slow response). ^(3)^  Transient sounds or L_max_^[[2]](#footnote-2)^ not exceeding 65dB (A weighted slow response).^(3)^  Staff, family and lounge areas: Continuous background and operation sounds not exceeding an hourly L_eq_ of 50dB and an hourly L10 of 55dB (both A weighted slow response).^(3)^  Transient sounds or L_max_ not exceeding 70dB (A weighted slow response) ^(3)^ |
| --- |
| 1. Adjustable volume control for announcing systems in sensitive areas ^(3)^ |
| 1. Acoustic seals on doors to ensure speech privacy and reduce intrusive sounds ^(3)^ |
| 1. Consideration of Speech Intelligibility ratings in infant, parent and staff areas^(3)^ |
| 1. Acoustically absorptive surfaces^(3)^ and walls build at obtuse angles to dissipate sound ^(4)^ |
| 1. Limiting glass to areas required for visualisation ^(3)^ |
| 1. Fire alarms in infant areas restricted to flashing lights.^(3)^ Telephones which use flashing lights to attract staff in clinical areas, rather than ring tones ^(4)^ |
| 1. Water supply and faucets chosen to minimise noise with instant warm water to reduce time water is running ^(3)^ |
| 1. Acoustic isolation of noise generating activities (multi-person work stations, linen carts etc.) and permanent equipment from infant areas ^(3)^ |
| 1. Vibration isolation pads under permanent equipment and appliances ^(3)^ |
| 1. Provision for post-construction evaluation of noise and vibration ^(3)^ |
| 1. Ceiling construction in adult sleeping and infant rooms should not be friable and have a noise reduction coefficient (NRC) of a minimum 0.90 for 80% of the entire surface area or an average NRC of 0.85 for the whole ceiling including solid and acoustically absorptive surfaces ^(3, 4)^ |
| 1. Enlisting the services of an acoustic engineer from project onset to post-construction validation ^(3)^ |

Table S2 Design specifications for optimum lighting

| 1. Ambient lighting levels in infant spaces shall be adjustable through a range of at least 10 to no more than 600 lux (approx. 1 to 60-foot candles), as measured on a horizontal plane at each bedside ^(3, 4)^ |
| --- |
| 1. Electric light sources shall have a CRI 8 of no less than 80, and a GA 9 of no less than 80 and no greater than 100 ^(3)^ |
| 1. Optical reﬂectors with a neutral ﬁnish ^(3)^ |
| 1. Avoidance of unnecessary ultraviolent or infrared radiation through appropriate lens or filters ^(3)^ |
| 1. No direct view of the electric light source or sun in the infant space and any lighting used outside the infant care area located so as to avoid any infant’s direct line of sight ^(3)^ |
| 1. Electric light sources that are supplied by 60 Hz alternating current should not ﬂicker more than a common 40 W incandescent light source. Speciﬁcally, the frequency and the depth of the light modulation produced by the source shall be no less than 120 Hz and no more than 13%, respectively ^(3)^ |
| 1. Adjustable (in terms if field size, intensity and direction), separate procedure lighting capable of providing not less than 2,000 lux at the plane of the infant bed, and framed so that no more than 2% of the light output of the luminaire extends beyond its illumination ﬁeld ^(3)^ |
| 1. Adequate illumination for staff support areas ^(3)^ |
| 1. At least one source of natural daylight should be visible from an infant space or room, either from an exterior window or exterior clerestory located at each infant space or in each room, or from an exterior (glazed) window or exterior clerestory in the staff work area adjacent to the infant space or room ^(3)^ |
| 1. Exterior window(s) should be situated at least 0.6 m (61cm in UK)^(4)^ away from any part of an infant’s bed and fitted with shading devices ^(2, 3)^ |

Table S3 Recommended sink design specifications

| Sink location   1. Every infant bed, whether in single or multiple rooms should be within six metres of a hands-free hand washing station.^(3, 4)^ 2. Stations should be no closer than 0.9m from an infant bed, clean supply storage or counter/work surface unless a splashguard is provided.^(3)^ |
| --- |
| Sink frequency   1. One large trough for every three infants is recommended.^(4)^ 2. Sufficient number and accessibility to encourage compliance with hand washing, balanced against the risk of excess sinks which are underused and result in stagnation.^(5)^ 3. Advice from the Infection Prevention and Control team should be sought.^(6)^ |
| Sink size   1. Large enough to control or contain splashing^(5)^ and designed to avoid standing or retained water.^(3)^ 2. Minimum sink dimensions are recommended to be 61x41x25 cm^3^ from the bottom of the sink to the top of its rim.^(3)^ |
| Taps   1. Aligned so that water does not flow directly into the drain and easily dismantled and removable for cleaning and disinfection.^(5, 6)^ 2. Sensor taps in augmented care units (such as NICU) should only be considered following risk assessment and not used where frequency of use is low.^(5)^ If required, sensor taps with automated programmable flushing capability could be considered^(5)^ but sensors should be fitted away from the tap.^(6)^ 3. Mixer taps with sealed cores and swan neck taps are not recommended.^(5)^ 4. Taps should be capable of including a point-of-use filter and have integral thermostatic control.^(6)^ |
| Finishes   1. Adjacent walls should be constructed from nonporous material.^(3)^ 2. Alcohol-based hand-rub dispensers should not be positioned where drips can fall on basins or taps.^(6)^ |

Table S4 Design features to enhance water safety

| 1. An Environmental Monitoring Committee and the Infection Prevention and Control team should be consulted during building ^(5)^ |
| --- |
| Hot and cold-water storage systems should be organised in a modular fashion in cases where future growth may increase demand. They should be designed to preserve water quality, to prevent microbial growth, eliminate or reduce the formation of aerosols, minimise corrosion and maintain internal surfaces in a clean condition ^(7)^ |
| 1. Provision of adequate access to components of the water system for cleaning and servicing ^(5)^ |
| 1. No decorative water features, wet evaporative cooling systems and coolers (including portable devices) are recommended ^(5)^ |
| 1. Intelligent water management systems and central absolute bacteria filters may mitigate risk ^(5)^ |
| 1. Materials, fixtures and fittings that support biofilm and micro-organism growth and colonisation should be avoided wherever possible ^(5)^ |
| 1. Tap rosettes are used to generate a straightened flow of water to enhance hand washing.^(5)^ However, a detailed inspection of rosettes taken from tap outlets in neonatal units in Northern Ireland during the outbreak of *Pseudomonas aeruginosa* found them to be heavily colonised, particularly complex design rosettes.^(8)^ A risk assessment should be carried out to determine if rosettes or flow straighteners should be in use ^(5, 6)^ |
| 1. Water distribution system components, such as flexible hoses, thermostatic mixing valves (TMVs), aerators and flow straighteners are associated with a high risk of contamination ^(5)^ |
| 1. A closed water system (with sterile water) must be used for infants who require therapeutic cooling ^(5)^ |
| 1. Total requirements for water supply and quality should be assessed and water systems appropriate to areas of accommodation allocated.^(7)^ When it is anticipated that areas of the building are likely to have different levels of occupancy and usage, consideration should be given to zoning of the water services to allow floors and areas of the building to be isolated and operated independently ^(7)^ |

Table S5 Design specifications for clinical, staff and family support areas

| Clinical support areas |
| --- |
| 1. General support space is required for clean and soiled utility/holding rooms and charting/staff work areas.^(3)^ |
| 1. At least 0.22m^3^ for each infant for secondary storage of syringe, needles, intravenous infusion sets and sterile trays.^(3)^ |
| 1. At least 1.7m^2^ of floor space allocated for equipment storage per infant in intermediate care and 2.8m^2^ for each infant bed in intensive care.^(3)^ |
| 1. Bedside storage should be at least 0.45m^3^ for each infant in the intermediate care area and 0.67m^3^ for each infant in the intensive care area. Beside storage should be designed for quiet operation.^(3)^ |
| Staff support areas |
| 1. May include lockers, lounge and private toilet facilities^(3)^ |
| 1. On call rooms should be provided, be immediately adjacent and be at least 10m^2^ with careful attention paid to soundproofing^(2, 4)^ |
| 1. If showers are provided they should not be fitted where they are likely to be used less than once a week^(5)^ |
| 1. Support space for ancillary services and administration^(3)^ |
| 1. Reception area is required with an open plan area of 15m^2(4)^ |
| 1. Unit office, of at least 20m^2^, large enough to accommodate four people with work surfaces on at least two walls^(4)^ |
| 1. Clinical manager and attending consultants offices should be located on the same floor^(2)^ |
| Family support areas |
| 1. Entrance area with space for coats or lockers and a prominent hand hygiene station^(2)^ |
| 1. Lounge area, nourishment area, lockable storage and toilet facilities^(3)^ |
| 1. A quiet room should be considered with the aim of providing distressed parents privacy. This should be close to toilet facilities and allow for exit without passing cot areas^(2)^ |

Table S6 Recommended space requirements for the NICU

| Individual infant space |
| --- |
| 1. Minimum 11.2m^2^ (or 12m^2^ ^(4)^) of clear floor space, excluding hand washing stations, columns and aisles, which may need to be increased with more complex care^(3)^ |
| 1. Sufficient furnishing to allow a parent to stay seated, reclining or fully recumbent at the bedside^(3)^ |
| 1. When access and support space is included with core cot space an area of 20m^2^ is recommended^(2)^ |
| Multi-cot areas |
| 1. Require a core clinical space of 13.5m^2^ (4.13m x 3.27m + 600mm access space) with a minimum distance of 2.4m between beds.^(2, 3)^ |
| Single-family rooms |
| 1. Minimum of 15.3m^2^ of clear floor area.^(3)^ |
| 1. Allow visual and speech privacy for the infant and family^(3)^ |
| 1. Capable of inclusion of a reclining chair for kangaroo care, a recumbent sleep surface, a desk or similar for writing/laptop use, at least four electrical outlets and no less than 0.2m^3^ storage space^(3)^ |
| 1. Designated staff space should include a work surface of no less than 0.6m^2^ with a charting surface no less than 0.3m^2^ and storage no less than 0.85m^3^.^(3)^ In practice, 20 to 24m^2^ is recommended space per cot^(4)^ |

Table S7 Design specifications to ensure NICU security

| 1. Clearly identified, single^(2)^ and access-controlled entrance and reception area for families^(2)^ |
| --- |
| 1. Limited number of exits and entrances^(3)^ |
| 1. Control stations located within close proximity and direct visibility to the entrance^(3)^ |
| 1. Security firm involvement in studying and planning the unit^(4)^ |

Table S8 Design specifications for NICU finishes

| Ceilings, walls and floors   1. Ceiling, wall, floor and furnishing finishes should have no or low volatile organic compounds (VOC) paints, coatings, materials and furnishings.^(3)^ 2. Wall and floor finishes should be designed for ease of cleaning, durability, minimise microorganism growth and have acoustic desirability.^(3)^ 3. Floor material should have a reflectance of no more than 40% and a gloss value of no more than 30 gloss units to minimise glare.^(3)^ |
| --- |
| Electrical outlets   1. At each bed there should be a minimum of 20 (20 to 24 in UK^(2, 4)^) simultaneously accessible electrical outlets, a minimum of three each of air, oxygen and vacuum gas outlets^(3, 4)^ as well as a dimmer switch, four computer ports and an emergency call bell.^(4)^ Access to more may be required for critically ill infants. |
| Ease of cleaning   1. Materials should also permit cleaning without the use of hazardous chemicals as it may not be possible to vacate rooms whilst cleaning.^(3)^ 2. Easily cleanable furnishings with few seams.^(3)^ |

Table S9 Design specifications to improve building sustainability

| 1. If possible, windows should have views of natural environments (trees, plants, gardens etc.) Within urban settings this may include planters.^(3)^ |
| --- |
| 1. A designated area for recyclable materials used in the NICU, measuring a minimum 1ft^2^ per patient bed, located outside the patient care area.^(3)^ |
| 1. Floor and wall surfaces, furnishings and ceiling finishes should be free of substances which are known to be carcinogenic, mutagenic and teratogenic or otherwise harm health.^(3)^ |
| 1. Specification of furnishings and materials from regional sources |
| 1. Furniture is recommended to be free from formaldehydes, polyvinyl chloride, halogenated flame retardants and perfluorinated compounds and sourced from sustainable forests.^(9)^ |
| 1. Facilitate healthy travel options for staff, patients, and visitors. Covered and secure bicycle parking should be provided.^(10)^ |

# Recommendations

| Infection prevention and control   1. A neutral pressure airborne infection isolation room, with a clear floor space of 14m^2^ should be provided in the NICU. This room should contain hand-washing facilities, space for storage, means of emergency communication and self-closing doors. 2. Every cot should be within 6m of a sink, but no closer than 0.9m. The number of sinks required will depend on the layout, but care should be taken to balance promotion of hand hygiene with the risks posed by stagnation from seldom used sinks. The infection control team should be involved in the planning phase. |
| --- |
| Single family rooms   1. Single family room design is recommended; however, consideration should be given to staff requirements if implemented. |
| Space requirements   1. An allocation of 20 to 24m^2^ per cot is recommended. In multi-cot areas, a core clinical space of 13.5m^2^ (4.13m x 3.27m + 600mm access space) per cot should be provided. Single cot areas require 20m^2^. This includes core cot, access and support space. Designated staff space should include a work surface of no less than 0.6m^2^ with a charting surface no less than 0.3m^2^ and storage no less than 0.85m^3^. |
| Location of the NICU   1. The NICU to be located as near as possible to theatres and labour wards. |
| Feeding facilities   1. Feeding facilities should include a central feeding preparation area. This should be distant to the bedside but close enough to allow ease of access for staff. 2. Mothers should be provided with a private breastfeeding space in which they feel a locus of control. If babies are cared for in multi-occupancy rooms, private space in which a mother can express milk should be provided. |
| Security within the NICU   1. The NICU should be served by a single, access-controlled entrance and reception area. |
| Finishes   1. Finishes should contain no or low VOC materials. They should combine ease of cleaning, potentially with hazardous materials, discourage microorganism growth and have acoustic desirability. Floor material should have a reflectance of no more than 40% and a gloss value of no more than 30 gloss units. 2. At each bed, there should be a minimum of 24 simultaneously accessible electrical outlets a minimum of three each of air, oxygen and vacuum gas outlets as well as a dimmer switch, four computer ports and an emergency call bell. |
| Support areas   1. Clinical support areas should be located near to care areas. There should be at least 0.22m^3^ for each infant for storage of disposable and smaller items. There should be at least 1.7m^2^ of floor space allocated for equipment storage per infant in intermediate care and 2.8m^2^ for each infant bed in intensive care. 2. One staff work station for every 6 to 18 beds with support space (which may occupy up to a third of the unit – ideally two stations for a 19-bed unit) including lockers, lounge, on-call rooms and private toilets. 3. A staff office at least 20m^2^. 4. Family support areas including a quiet room, lounge area, lockable storage and toilets. |
| Sustainability   1. A designated area for recyclable materials used in the NICU, measuring a minimum 1ft^2^ per patient bed, located outside the patient care area. 2. Sustainable measures including designated recycling area, locally sourced materials from sustainable sources. Products containing formaldehydes, polyvinyl chloride, halogenated flame retardants and perfluorinated compounds should be avoided. |

1. Shekelle PG, Woolf SH, Eccles M, Grimshaw J. Developing clinical guidelines. Western Journal of Medicine. 1999;170(6):348-51.

2. Department of Health. Health Building Note 09-03: Neonatal Units. United Kingdom: Department of Health, 2013.

3. White RD, Smith JA, Shepley MM. Recommended standards for newborn ICU design, eighth edition. Journal of Perinatoloy. 2013;33 Suppl 1:S2-16.

4. Laing I, Ducker T, Leaf A, Newmarch P. Designing a Neonatal Unit - Report for the British Association of Perinatal Medicine. British Association of Perinatal Medicine, 2004.

5. Prevention and Control of Infection from Water Systems in Healthcare Facilities Sub-Committee of the HPSC Scientific Advisory Committee. Guidelines for the Prevention and Control of Infection from Water Systems in Healthcare Facilities. Dublin: HPSC, 2016.

6. HFS HPS and Pseudomonas aeruginosa and Water (Scotland) Group. Guidance for neonatal units (NNUs) (levels 1, 2 & 3), adult and paediatric intensive care units (ICUs) in Scotland to minimise the risk of Pseudomonas aeruginosa infection from water [www.hps.scot.nhs.uk/haiic/ic/resourcedetail.aspx?id=176:](http://www.hps.scot.nhs.uk/haiic/ic/resourcedetail.aspx?id=176:) Health Protection Scotland; 2017.

7. Health Protection Surveillance Centre. National Guidelines for the Control of Legionellosis in Ireland. Dublin: HPSC, 2009.

8. The Regulation and Quality Improvement Authority. Independent Review of Incidents of Pseudomonas aeruginosa Infection in Neonatal Units in Northern Ireland Belfast: RQIA, 2012.

9. National Health Sustainability Office. Specifying Healthy Healthcare Interiors for HSE Capital Projects In: Health Service Executive, editor.: HSE; 2013.

10. HSE Estates. Sustainable Healthcare Building Guidelines - Specification, design, construction and refurbishment of Healthcare Buildings Ireland: HSE.

1. L_10_ is the sound level that could be exceeded only 10% of each hour [↑](#footnote-ref-1)
2. Maximum sound pressure level associated with an individual noise event [↑](#footnote-ref-2)
